# The influence of religion on physicans` and nurses` attitudes toward medical cannabis in Northern Israel

**DOI:** 10.1186/s42238-025-00331-6

**Published:** 2025-09-29

**Authors:** Loay Zaknoun, Salman Zarka, Ygal Plakht, Orli Grinstein-Cohen

**Affiliations:** 1 Nursing Department, Faculty of Health Sciences, Ben-Gurion University of the Negev, Beer-Sheva, Israel; 2 Ziv Medical Center, Safed, Israel

**Keywords:** Medical cannabis, Religion, Physicians, Nurses, Attitudes

## Abstract

**Background:**

The use of medical cannabis has been increasing significantly worldwide, including in Israel, a country characterized by substantial religious diversity. This study examines the influence of religion on physicians’ and nurses’ attitudes toward medical cannabis use, focusing on four primary religions in Northern Israel: Judaism, Islam, Christianity, and the Druze faith.

**Methods:**

A descriptive cross-sectional study was conducted at the Ziv Medical Center in Northern Israel, involving 395 physicians and nurses. Participants completed a structured questionnaire based on a modified version of the Medical Marijuana Questionnaire (MMQ), assessing their attitudes toward the medical benefits and risks of cannabis. Data were analyzed using ANCOVA and mixed-design ANCOVA models, controlling for covariates including age, gender, religiosity, profession, educational level, and exposure to cannabis use among acquaintances.

**Results:**

Statistical analyses revealed significant differences in attitudes toward medical cannabis across religious groups. After adjusting for demographic and background variables, Jewish and Christian participants reported significantly more favorable attitudes regarding the medical benefits of cannabis, while Muslim and Druze participants emphasized its associated risks. A significant interaction was also found between religious affiliation and attitude type (benefits vs. risks), indicating that religious affiliation moderated the relative evaluation of cannabis’s therapeutic potential versus its harms. These findings suggest that each religion’s unique cultural and ethical frameworks shaped participants’ attitudes. The more permissive attitudes observed among Jewish and Christian participants reflect religious principles that emphasize alleviating suffering, while the cautious attitudes of Muslim and Druze participants align with stricter interpretations of religious guidelines.

**Conclusions:**

This study identified significant differences in attitudes toward medical cannabis among physicians and nurses from different religious backgrounds in Northern Israel. Jewish and Christian participants expressed more favorable views regarding its medical use, while Muslim and Druze participants exhibited more cautious attitudes. These findings underscore the need for culturally and religiously tailored education and policies to facilitate the integration of medical cannabis into clinical practice.

## Background

Cannabis has been used by humans for millennia, dating back to the earliest civilizations. Historically, its use was as commonplace as tobacco, alcohol, and caffeine (Crocq 2020). Archaeological and textual evidence suggests that cannabis played a role in both daily life and ritual practices across cultures (Maguire 2020; Heilig 2011). Its therapeutic properties were known and utilized long before the emergence of modern pharmacology. Ancient Chinese, Indian, and Middle Eastern medical traditions incorporated cannabis to alleviate pain, inflammation, and other ailments (Balant et al. 2021; Pisanti et al., 2018). In recent years, the use of medical cannabis has increased significantly worldwide (United Nations, 2022).

While legal and clinical aspects of medical cannabis have been extensively studied, its sociocultural dimensions - especially religious influences - remain under-explored. Religion is not only a major determinant of individual beliefs and social norms (Mazur 2020), but also a central force in shaping health-related behaviors and clinical decision-making (Borges et al. 2021; Koenig et al., 2012). Several studies have confirmed a link between religious affiliation and attitudes toward cannabis use (Clobes and Gagnon 2022; Felson et al. 2019; Ferrara 2021; Gritsenko et al. 2020; Nie and Yang 2018; Rafei et al. 2023; Siddiqui et al. 2022). These theoretical frameworks provide a foundation for understanding the observed differences in physicians’ and nurses’ attitudes across religious groups.

Despite the growing scholarly interest in religious perspectives on cannabis, empirical research directly examining the views of religious leaders remains scarce. Nevertheless, religious authorities are widely recognized as influential opinion leaders within their communities, particularly on moral and health-related issues. Their interpretations often guide believers’ attitudes toward medical interventions, including cannabis use, and may either reinforce or moderate formal doctrinal positions (Koenig et al., 2012).

Each religion approaches cannabis use through its unique theological and moral frameworks. In Judaism, references to cannabis can be traced back as early as the Exodus (ca. 1220 BCE). Polish anthropologist Sula Benet argued that cannabis appears in the Hebrew and Aramaic Bible and the Talmud, often in the context of religious rituals (Koltai et al. 2022; Pisanti et al., 2018). In modern Jewish discourse, rabbinical rulings have drawn a clear distinction between medical use, which is generally permitted, and recreational use, which is largely prohibited. In Islam, cannabis use in the Middle East began approximately two centuries after the Prophet Muhammad’s death (Abdulrahman 2024; Ghiabia et al., 2018). During the Prophet’s time, cannabis (known as “hashish” or “grass” in Arabic) was not recognized, and thus no prohibition is mentioned in the Quran (Marino 2022; Nahas 1982; Pisanti et al., 2018). In modern Islamic contexts, recreational use of cannabis is strictly forbidden, while its medical use is permitted (Shirah and Ahmed 2020). In Christianity, recreational use of cannabis is generally opposed, while its medical use is permitted, as indicated in the *Catechism of the Catholic Church*, which identifies drug use as sinful unless intended for medical purposes (Dhanasekaran et al. 2022). The Druze religion is a closed and secretive philosophical religion with doctrines accessible only to a select group of religious leaders known as the *Uqqal* (“wise men”). A broader group of lay followers, the *Juhhal* (“ignorant ones”), remain unfamiliar with the deeper tenets of the faith (Weisber 2022). Despite extensive searches, no official stance regarding medical or recreational cannabis has been identified.

Beyond religious affiliation, other factors such as the level of religiosity, educational background, gender, and personal familiarity with cannabis users may also influence attitudes toward its medical use (Amoateng and Bahr 1986; Edelstein et al. 2020; Cameron et al. 2023; Cuttler et al. 2016; Desmond et al. 2008; HaGani et al. 2021; Marsiglia et al. 2005; Rafei et al. 2023; Sokratous et al. 2023). Nonetheless, religion remains a dominant cultural force shaping public and professional perspectives on health interventions, especially those involving controversial substances (Mazur 2020).

In Israel, due to changes in governmental policies, there has been a notable rise in medical cannabis use, as well as in the number of licenses issued to oncology patients, which increased from 6.8 to 9.4% between 2014 and October 2018 (Sznitman 2020). Northern Israel provides a unique setting for exploring these dynamics. Its population is highly diverse and multicultural, comprising significant communities of Jews, Muslims, Christians, and Druze. This religious heterogeneity allows for a comparative analysis of attitudes across different belief systems within a shared geographical and healthcare context. The Ziv Medical Center, located in the Galilee region, serves as a microcosm of this diversity, in terms of both its patients and its healthcare staff. Despite the growing significance of medical cannabis, no empirical study has yet examined how religion influence healthcare professionals’ attitudes toward its use in Northern Israel. Moreover, the attitudes of Druze professionals - who represent a substantial portion of the regional healthcare workforce - remain entirely unexamined in the scientific literature.

This study addresses these gaps by investigating the relationship between religion and attitudes toward medical cannabis among physicians and nurses in Northern Israel. It is the first study to explore this topic in a multicultural Israeli context and to include Druze and Christian participants as a distinct religious group. By doing so, the study aims to contribute to culturally sensitive healthcare policy and education. This study is guided by two main hypotheses: (1) There will be differences in attitudes and beliefs about medical cannabis based on religious affiliation. (2) Religious affiliation will moderate the differences in attitudes and beliefs toward medical cannabis.

## Methods

### Study setting and data collection

A cross-sectional study was conducted at the Ziv Medical Center in Northern Israel, which is the sole facility in the region, benefiting from the multicultural richness of the northern area and the diverse religions and ethnic communities in its vicinity. This diversity is reflected in the composition of both its workforce and its patient population. The data collection took place between June and September 2024. All procedures were performed in compliance with relevant laws and institutional guidelines and have been approved by the institutional ethics committee (ZIV-0037-24). Upon receiving this approval, the researcher coordinated with department directors and head nurses to gain access to departmental team meetings. During these meetings, the researcher explained the study’s purpose, emphasized its anonymity, explained the concept of informed consent, and underscored the voluntary nature of questionnaire completion. The research questionnaires were then distributed, and the researcher left the meeting. To enhance anonymity, participants completed the questionnaires independently and deposited them in a designated collection box. A total of 550 questionnaires were distributed, of which 395 were returned, yielding a participation rate of 71.5%.

### Measures

#### Attitudes and beliefs about medical cannabis

Attitudes and beliefs about medical cannabis were assessed using a modified version of the Medical Marijuana Questionnaire (Chan et al. 2017). Eight items from the original questionnaire were selected for the present study. An example item is, “Physicians should recommend cannabis as a medical therapy”. Participants were asked to rate their agreement on a six-point Likert scale (1 = strongly disagree to 6 = strongly agree). In the original study, Cronbach’s α ranged from 0.82 to 0.91 (Chan et al. 2017). An Exploratory Factor Analysis (EFA) with principal component extraction and oblimin rotation was applied to the eight items. Based on Kaiser’s criterion (eigenvalues greater than one; Kaiser 1960), the scree plot, parallel analysis, and Velicer’s minimum average partial test (O’Connor, 2000), two factors emerged, explaining about 67.8% of the variance. Item loadings ranged from 0.42 to 0.94. Factor 1 reflected beliefs about the medical benefits of cannabis, while Factor 2 captured beliefs about its medical risks. Internal consistency was satisfactory (Table 1). Mean scores were calculated for each of the two factors, with higher scores indicating stronger attitudes and beliefs. The two factors were negatively correlated, *r*(393) = − 0.37, *p* < .001.

**Table 1:** Results of exploratory factor analysis on the attitudes and beliefs about medical cannabis items

Item	Medical benefits	Medical risks
Physicians should recommend cannabis as a medical therapy	0.94	
There are significant physical health benefits to using medical cannabis	0.93	
I would recommend a patient to use medical cannabis	0.91	
There are significant mental health benefits to using medical cannabis	0.79	
Cannabis should be legalized also for recreational use	0.42	
Using cannabis poses serious physical health risks		0.87
Using cannabis poses serious mental health risks		0.83
Cannabis can be addictive		0.74
Eigenvalue	3.89	1.53
% of variance explained	48.7%	19.1%
Cronbach’s α	0.85	0.76

*Note. N =* 395. Factor loadings above 0.40 are shown

### Data analysis

Preliminary analysis included comparing background variables across religious affiliation. One-way analyses of variance (ANOVAs) followed by Bonferroni-adjusted post-hoc tests were employed for continuous variables, while the chi-square (χ^2^) tests of independence were used for categorical variables. Significant background variables were controlled in hypothesis testing (i.e. age, gender, religiosity, profession, education, and medical or recreational cannabis usage by family or friends). The present study tested two hypothesis: (1) There will be differences in attitudes and beliefs about medical cannabis based on religious ​affiliation. (2) Religious affiliation will moderate the differences in attitudes and beliefs toward medical cannabis. For Hypothesis 1, one-way analyses of covariance (ANCOVA) followed by Bonferroni-adjusted post-hoc tests assessed differences in attitudes and beliefs about medical cannabis by religious preference. For Hypothesis 2, a mixed-design ANCOVA examined differences between medical benefits and risks across religious preferences (difference-in-difference). Statistical Package for the Social Sciences (SPSS) version 29 (IBM Corporation, Armonk, NY) was used, with α = 0.05 (2-tailed) for all tests.

## Results

The aim of this study was to examine how religious affiliation influences physicians’ and nurses’ attitudes and beliefs toward medical cannabis, specifically focusing on perceived medical benefits and risks. A cross-sectional sample of 395 physicians and nurses categorized by religious affiliation (Jewish, Muslim, Christian, Druze) was analyzed (Table 2). Demographic and background variables were compared to identify any significant group differences. The results are presented in three parts: preliminary analysis of background variables, hypothesis-testing regarding differences by religion, and analysis of interaction effects between attitude type and religious affiliation.

**Table 2:** Sample characteristics

Variable	Total	Religious affiliation				Difference test
		Jewish	Muslim	Christian	Druze	
	(*N* = 395)	(*n* = 145)	(*n* = 98)	(*n* = 74)	(*n* = 78)	
Age, *M (SD)*	39.77 (10.65)	43.89_a_ (10.86)	33.95_b_ (6.39)	39.82_c_ (10.54)	39.38_c_ (11.29)	*F*(3, 391) = 19.41, *p* < .001, η² = 0.13
Seniority, *M (SD)*	13.09 (10.99)	16.34_a_ (12.35)	8.44_b_ (6.63)	13.10_a, c_ (10.59)	12.44_a, b,c_ (10.88)	*F*(3, 350) = 9.59, *p* < .001, η^2^ = 0.08
Gender, *n* (%)						χ²(3, *N* = 395) = 55.35, *p* < .001
Male	172 (43.5)	28_a_ (19.3)	57_b_ (58.2)	40_b_ (54.1)	47_b_ (60.3)	
Female	223 (56.5)	117_a_ (80.7)	41_b_ (41.8)	34_b_ (45.9)	31_b_ (39.7)	
Marital status, *n* (%)						χ²(3, *N* = 395) = 3.04, *p* = .386
Married or in a relationship	314 (79.5)	119 (82.1)	75 (76.5)	55 (74.3)	65 (83.3)	
Other	81 (20.5)	26 (17.9)	23 (23.5)	19 (25.7)	13 (16.7)	
Religiosity, *n* (%)						χ²(6, *N* = 395) = 34.26, *p* < .001
Secular	162 (41.0)	66_a_ (45.5)	23_b_ (23.5)	36_a_ (48.6)	37_a_ (47.4)	
Traditional	168 (42.5)	46_a_ (31.7)	52_b_ (53.1)	32_a, b_ (43.2)	38_a, b_ (48.7)	
Religious	65 (16.5)	33 (22.8)	23_b_ (23.5)	6_b_ (8.1)	3_b_ (3.8)	
Education, *n* (%)						χ²(3, *N* = 395) = 16.28, *p* < .001
Diploma or B.A.	188 (47.6)	88_a_ (60.7)	41_b_ (41.8)	27_b_ (36.5)	32_b_ (41.0)	
M.D., M.A. or higher	207 (52.4)	57_a_ (39.3)	57_b_ (58.2)	47_b_ (63.5)	46_b_ (59.0)	
Profession, *n* (%)						χ²(3, *N* = 395) = 24.82, *p* < .001
Physician	137 (34.7)	28_a_ (19.3)	45_b_ (45.9)	29_b_ (39.2)	35_b_ (44.9)	
Nurse	258 (65.3)	117_a_ (80.7)	53_b_ (54.1)	45_b_ (60.8)	43_b_ (55.1)	
Family member(s) or friend(s) who use medical cannabis						χ²(3, *N* = 395) = 27.83, *p* < .001
Yes	129 (32.7)	69_a_ (47.6)	16_b_ (16.3)	23_a, b_ (31.1)	21_b_ (26.9)	
No	266 (67.3)	76_a_ (52.4)	82_b_ (83.7)	51_a, b_ (68.9)	57_b_ (73.1)	
Family member(s) or friend(s) who use recreational cannabis						χ²(3, *N* = 395) = 21.60, *p* < .001
Yes	155 (39.2)	67_a_ (46.2)	19_b_ (19.4)	33_a_ (44.6)	36_a_ (46.2)	
No	240 (60.8)	78_a_ (53.8)	79_b_ (80.6)	41_a_ (55.4)	42_a_ (53.8)	

Note. Data were missing for 41 cases in the question about seniority. Categories with different subscript letters differ significantly from each other at the 0.05 level

### Preliminary analysis

We first identified potential covariates for subsequent analysis by examining differences in background characteristics based on religious affiliation. The analyses revealed that all group comparisons were statistically significant except for marital status (*p = .386*) (Table 2). Jewish participants were older, predominantly female, more commonly nurses, and had lower levels of education than participants from other religious groups. Additionally, Jewish participants were more likely to report having family members or friends who used medical cannabis. Muslim participants were less likely to identify as secular relative to other religious groups and had the lowest seniority compared to Jewish and Christian participants, although their seniority was not significantly different from that of Druze participants. Due to the strong correlation between age and seniority (*r = .92*, *p < .001*), seniority was excluded from subsequent analyses to avoid multicollinearity.

### Hypothesis 1: differences in attitudes and beliefs based on religious affiliation

We hypothesized that physicians` and nurses’ attitudes and beliefs regarding medical cannabis would vary significantly according to religious affiliation (Hypothesis 1). To evaluate this, two one-way ANCOVAs were used to assess differences in perceived medical benefits and medical risks of cannabis (see Table 3). The analysis controlled for age, gender, religiosity, profession, education, and cannabis use among family or friends. A significant effect of religious affiliation, *F*(3, 383) = 26.88, *p* < .001, partial η² = 0.17 on perceived medical benefits was indicated. Christian (M = 4.44) and Jewish (M = 4.38) participants reported significantly higher adjusted mean scores compared to Muslim (M = 3.58) and Druze (M = 3.47) participants. For perceived medical risks, the ANCOVA also showed a significant group effect, *F*(3, 383) = 4.38, *p* = .005, partial η² = 0.03. Druze participants reported the highest adjusted mean for perceived risks (M = 4.60), significantly greater than Christian and Jewish participants, though not significantly different from Muslim participants. These findings support Hypothesis 1, indicating that religious affiliation is associated with differences in attitudes and beliefs toward medical cannabis. Specifically, Christian and Jewish participants were more likely to endorse its medical benefits, whereas Muslim and Druze participants emphasized its potential risk.

**Table 3:** Means, adjusted means, standard deviations, and one-way ANCOVA in measures of attitudes and beliefs about medical cannabis

**Variable**	Religious affiliation				**Difference test**
	Jewish	Muslim	Christian	Druze	
	(*n* = 145)	(*n* = 98)	(*n* = 74)	(*n* = 78)	
	***M*/Adj.*M*** (***SD***)	***M*/Adj.*M*** (***SD***)	***M*/Adj.*M*** (***SD***)	***M*/Adj.*M*** (***SD***)	
Medical benefits	4.45/4.38_a_ (0.81)	3.46/3.58_b_ (0.98)	4.53/4.44_a_ (0.67)	3.54/3.47_b_ (1.06)	*F*(3, 383) = 26.88, *p* < .001, η²_p_ = .17
Medical risks	4.13/4.22_a_ (1.00)	4.48/4.35_ab_ (0.83)	4.07/4.09_a_ (0.93)	4.59/4.60_b_ (0.87)	*F*(3, 383) = 4.38, *p* = .005, η²_p_ = .03

Note. All results are controlled for age, gender, religiosity, profession, education, and medical or recreational cannabis usage by family or friends. Adj. = adjusted

### Hypothesis 2: religious affiliation moderates the differences between perceived benefits and risks

We further hypothesized that religious affiliation would moderate the differences between perceived benefits and risks (Hypothesis 2).

To examine this, a mixed-design ANCOVA was conducted. The within-subjects factor was attitude type (medical benefits vs. medical risks), the between-subjects factor was religious affiliation, and the model controlled for the same covariates as in the previous analysis.

**Fig. 1:**
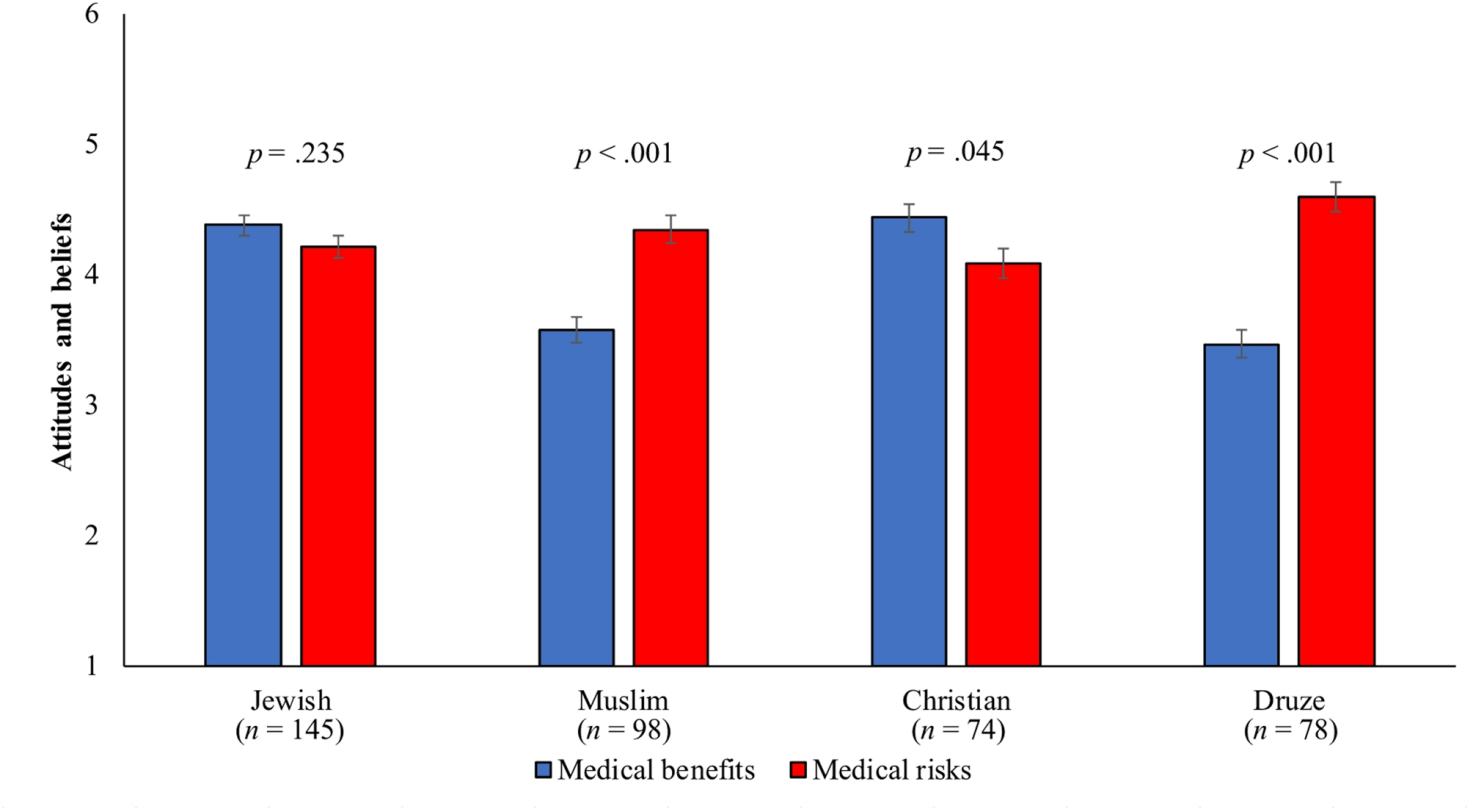
Differences in attitudes and beliefs about medical cannabis based on religious affiliation. Data concerning attitudes towards medical cannabis were collected from 395 physicians and nurses (Table 2) as part of a cross-sectional study conducted at the ZIV Medical Center in Northern Israel. Data were used in a mixed-design ANCOVA to characterize attitudes toward medical cannabis and categorized by religious affiliation (Jewish, Muslim, Christian, Druze) with respect to perceived medical benefits and medical risks. The analysis was controlled for age, gender, religiosity, profession, education, and medical or recreational cannabis usage by family or friends. Attitudes were scored on a 6-point Likert scale (1 = strongly disagree, 6 = strongly agree)

The analysis revealed a significant interaction between attitude type and religious affiliation, *F*(3, 383) = 19.26, *p* < .001, η² = 0.13 (Fig. 1).

Post-hoc comparisons revealed the following pattern:

- Christian participants rated medical benefits significantly higher than medical risks.
- Muslim and Druze participants rated medical risks higher than benefits.
- Jewish participants showed no significant difference between benefits and risks.

These findings support Hypothesis 2, suggesting that religious affiliation both influences the overall evaluation of medical cannabis and moderates the relative weighting of perceived benefits versus risks.

## Discussion

This study examines the impact of religion on shaping the attitudes of physicians and nurses toward medical cannabis in Northern Israel. The region’s unique cultural and religious diversity provides a valuable context for understanding how faith-based values influence attitudes regarding the perceived benefits and risks of medical cannabis use. Through rigorous statistical controls, the analysis isolates the effects of religion, offering important insights into the interplay between personal beliefs and professional responsibilities.

### Religion as a key determinant of attitudes towards medical cannabis

The findings indicate significant differences in attitudes across the four major religious groups in Northern Israel: Judaism, Christianity, Islam, and the Druze faith. Jewish and Christian participants demonstrated higher recognition of the medical benefits of cannabis, whereas Muslim and Druze participants emphasized its associated risks. These findings suggest that religious affiliation may not merely act as a direct predictor of attitudes toward medical cannabis, but also moderate the evaluative process through which individuals balance perceived medical benefits against perceived risks of cannabis use.

In Judaism, the principle of “*pikuach nefesh*” (saving a life) serves as a guiding ethical framework, permitting interventions that alleviate suffering, even if they involve substances typically prohibited. Rabbinical rulings have often supported the use of prohibited substances in medical treatments, including cannabis, when it aligns with these principles (Bleich 2022; Pisanti et al., 2018). This permissive stance likely contributes to the positive attitudes observed among Jewish participants.

Christianity also supports the use of potentially harmful substances (such as drugs) for medical purposes when consistent with ethical and moral guidelines. The Catechism of the Catholic Church explicitly permits medical applications of substances that could be harmful, as long as they are used to alleviate suffering or illness (Catechism, 2291; Dhanasekaran et al. 2022). These shared ethical values provide a foundation for the positive attitudes among Christian participants.

Conversely, Islamic teachings impose stricter controls on substances considered intoxicants, with exceptions allowed only for legitimate medical needs (Nassif 2021; Qatanani et al. 2021). The Quran emphasizes the importance of protecting the body from harm, a principle that informs the cautious attitudes observed among Muslim participants (Shirah and Ahmed 2020). Modern Islamic rulings often require a higher burden of proof to justify medical cannabis use, reinforcing this cautious approach.

Druze participants reported the highest levels of concern regarding the risks of medical cannabis. The Druze faith, characterized by its conservative and secretive ethos, lacks explicit doctrinal guidance on cannabis use. As a result, Druze healthcare may rely on cultural norms that emphasize caution in the absence of clear religious directives (Weisber 2022). This highlights the influence of community and cultural factors alongside formal religious teachings.

These findings align with broader theoretical frameworks that conceptualize religion as a key sociocultural determinant of health-related behaviors. As Koenig and colleagues (2012) and Borges and colleagues (2021) argue, religious affiliation influences not only personal values but also clinical decision-making, particularly in ethically complex contexts such as medical cannabis. In this study, both doctrinal principles and cultural expressions of religion shaped participants’ attitudes. This dynamic is especially salient in multicultural regions like Northern Israel, where healthcare professionals must reconcile personal beliefs with professional obligations across diverse religious landscapes.

### Implications for policy, practice and public health

The Findings of this study carry important implications for the development of culturally sensitive policies and educational initiatives in healthcare systems serving religiously diverse populations. In regions such as Northern Israel, where religious affiliation deeply shapes ethical reasoning and health-related behaviors, the integration of medical cannabis into clinical practice requires a nuanced understanding of doctrinal positions, cultural norms, and professional responsibilities.

At the policy and clinical practice level, decision-makers must account for the ethical frameworks and religious doctrines that influence physicians` and nurses` acceptance of medical cannabis. Policies that acknowledge and respect these religious sensitivities are more likely to gain the trust of healthcare professionals and promote equitable access to cannabis-based treatments. Institutional guidelines should therefore be developed in consultation with religious authorities and cultural representatives, particularly in settings where faith-based values play a central role in professional identity and decision-making.

To support implementation, educational programs for healthcare providers must address both the clinical evidence and the ethical tensions surrounding medical cannabis. Such training should incorporate discussions on aligning therapeutic cannabis use with religious principles, emphasizing its controlled and evidence-based application for alleviating suffering. This approach can help professionals navigate perceived conflicts between personal beliefs and clinical obligations, thereby enhancing ethical confidence and treatment consistency.

From a broader public health perspective, effective communication strategies must also engage religious frameworks to foster societal acceptance of medical cannabis. In communities where religious values shape public attitudes, health promotion efforts should be designed to resonate with spiritual and moral worldviews. Messaging that highlights the therapeutic intent, medical supervision, and ethical legitimacy of medical cannabis use can reduce stigma and support informed patient decision-making.

Collaboration with religious leaders is particularly valuable in this regard. As respected moral authorities, they can play a pivotal role in reshaping public attitudes and legitimizing medical cannabis within their communities. Their involvement can also bridge gaps between scientific evidence and faith-based skepticism, contributing to a more inclusive and ethically grounded discourse on cannabis use in medicine.

Ultimately, the study`s insights are relevant beyond Northern Israel. They offer a model for integrating medical cannabis into healthcare systems that serve diverse religious populations, demonstrating how ethical pluralism and clinical pragmatism can be effectively reconciled in complex sociocultural environments.

### Limitations

While this study provides valuable insights, it has several limitations that warrant further investigation. The cross-sectional design limits the ability to infer causality, and the reliance on self-reported data introduces the potential for response bias. Additionally, The study was conducted among physicians and nurses at a single medical center located in a peripheral region of Israel, where sociocultural and religious dynamics may differ from those in other parts of the country, especially in central areas.

Future research should adopt longitudinal designs to examine how attitudes toward medical cannabis evolve over time, particularly in response to shifts in religious discourse or policy environments. Replicating the study across additional geographic regions - especially in central Israel - and including other healthcare providers could provide a more comprehensive understanding.

Qualitative studies, such as in-depth interviews with religious leaders and focus groups, could provide richer insights into the complex factors shaping attitudes. These approaches could explore how personal and professional experiences intersect with religious beliefs to influence perceptions of medical cannabis. Additionally, they could help elucidate whether the method of medical cannabis use affects attitudes. Further investigation into the Druze population is particularly important, given the absence of explicit doctrinal guidance on cannabis use within this community.

### Religiosity and implications for future research

While the present study focused on religious affiliation, it did not examine religiosity as a primary independent variable, due to the relatively small number of participants who identified as religious. Nonetheless, prior studies suggest that individuals with higher levels of religiosity tend to view medical cannabis through a moral lens shaped by their adherence to religious doctrines. This perspective emphasizes ethical caution and aligns with global trends linking higher religiosity to more conservative attitudes toward health-related behaviors (Amoateng and Bahr 1986; Edelstein et al. 2020; Findley et al. 2021; Nie and Yang 2018).

Given these insights, future research should examine how varying degrees of religiosity interact with religious affiliation to shape physicians’ and nurses’ attitudes toward medical cannabis. Such investigations would be particularly valuable in multicultural contexts like Northern Israel, where clinical practice intersects with diverse religious and ethical frameworks.

## Conclusions and recommendations

This study contributes to interdisciplinary discourse on religion and health policy by offering empirical evidence on how religious affiliation shapes physicians’ and nurses’ attitudes toward medical cannabis within a multicultural healthcare context. To the best of our knowledge, this is the first known investigation to specifically examine the attitudes of the Druze population toward cannabis, thereby filling a critical gap in the literature concerning underrepresented religious minorities. The inclusion of four distinct religious groups within a shared healthcare setting offers a rare opportunity to compare intra-professional variation in religious reasoning. As the global integration of medical cannabis advances, the implications of this study extend beyond the Israeli context, underscoring the need for culturally competent policies, education programs, and clinical guidelines that account for pluralistic religious orientations.

## Data Availability

The data that support the findings of this study are available upon request from the corresponding author.

## Abbreviations

ANOVA: Analysis of variance
ANCOVA: Analysis of covariance
EFA: Exploratory factor analysis
